# Ceramic-Chromium Hall Sensors for Environments with High Temperatures and Neutron Radiation

**DOI:** 10.3390/s21030721

**Published:** 2021-01-21

**Authors:** Slavomir Entler, Zbynek Soban, Ivan Duran, Karel Kovarik, Karel Vyborny, Josef Sebek, Stana Tazlaru, Jan Strelecek, Petr Sladek

**Affiliations:** 1 Institute of Plasma Physics of CAS, Za Slovankou 3, 182 00 Prague, Czech Republic; duran@ipp.cas.cz (I.D.); kovarik@ipp.cas.cz (K.K.); sladek@ipp.cas.cz (P.S.); 2 Institute of Physics of CAS, Cukrovarnicka 10/112, 162 00 Prague 6, Czech Republic; soban@fzu.cz (Z.S.); vybornyk@fzu.cz (K.V.); 3 Institute of Physics of CAS, Na Slovance 1999/2, 182 21 Prague 8, Czech Republic; sebek@fzu.cz; 4 Faculty of Mathematics and Physics, Charles University, Ke Karlovu 5, 121 16 Prague 2, Czech Republic; stana.tazlaru@morp.cz (S.T.); jan.strelecek@morp.cz (J.S.)

**Keywords:** Hall sensors, metal, chromium, nanolayer, high temperature, radiation, resistant, nuclear, fusion, DEMO

## Abstract

Ceramic-chromium Hall sensors represent a temperature and radiation resistant alternative to Hall sensors based on semiconductors. Demand for these sensors is presently motivated by the ITER and DEMO nuclear fusion projects. The developed ceramic-chromium Hall sensors were tested up to a temperature of 550 °C and a magnetic field of 14 T. The magnitude of the sensitivity of the tested sensor was 6.2 mV/A/T at 20 °C and 4.6 mV/A/T at 500 °C. The sensitivity was observed to be weakly dependent on a temperature above 240 °C with an average temperature coefficient of 0.014%/°C and independent of the magnetic field with a relative average deviation below the measurement accuracy of 0.086%. A simulation of a neutron-induced transmutation was performed to assess changes in the composition of the chromium. After 5.2 operational years of the DEMO fusion reactor, the transmuted fraction of the chromium sensitive layer was found to be 0.27% at the most exposed sensor location behind the divertor cassette with a neutron fluence of 6.08 × 10^25^ n/m^2^. The ceramic-chromium Hall sensors show the potential to be suitable magnetic sensors for environments with high temperatures and strong neutron radiation.

## 1. Introduction

Hall sensors represent a currently well-known industrial technology widely used for detecting speed, proximity, position, and electric current in many areas of automation, robotics, automotive and electrical engineering [1]. The Hall voltage is measured on a thin layer of semiconductor material, usually equipped with four contacts for supply and voltage sensing. The most commonly used semiconductors for these sensors are InSb, InAs, GaAs, and Si [1]. Semiconductors offer high sensitivity to magnetic fields, but they degrade in environments with high temperatures or intense radiation [2,3,4,5]. Because the Hall effect exists in metals and semi-metals as well as in semiconductors, it is possible to produce the sensitive layer from semi-metal whose electronic band structure resembles that of a semiconductor while maintaining the finite density of states at the Fermi level even in the purest systems [6]. These features render semi-metals, such as bismuth or antimony, significantly more resistant to temperature and radiation compared to semiconductors while the sensitivity remains higher than in metals. The sensitivity of the Hall sensors with the sensitive layer made of metal (hereafter metal Hall sensors) is many orders of magnitude lower than that of semiconductor Hall sensors. Table 1 presents Hall coefficients characterizing the Hall effect in some metals, semi-metals, and the InSb semiconductor (note that the InSb Hall coefficient strongly depends on the doping and preparation conditions).

Due to the metal Hall sensors’ low output signal related to the low sensitivity, the influence of many spurious effects such as induction, crosstalk, or leakage currents increases. The temperature-dependent offset and the interfering planar Hall voltage are also a challenge. Therefore, the metal Hall sensors need sophisticated signal conditioning and processing to make Hall sensor output usable for applications. The sensor signal must be processed by advanced methods such as synchronous detection or current spinning. This process can be performed by a Hall sensor controller, which supplies the sensor and amplifies the Hall voltage [10].

In addition to sensitive material, all other materials used within the sensor body must be also suitable for environments with high temperatures and neutron radiation. Materials that lose the ability of electrical insulation or are dimensionally unstable under operating conditions cannot be used. Many ceramic materials are suitable for high temperatures, however, not every ceramic material is fit for high-temperature electrical insulation.

High temperature and radiation resistant Hall sensors have not been widely used so far. However, there has been a demand that recently emerged in connection to the ongoing development of advanced nuclear fusion reactors such as ITER [11] and demonstration fusion power plants dubbed DEMO [12]. In the fusion power reactors, the Hall sensors will be a part of the reactor control system [13].

Demand for Hall sensors resistant to high temperature and radiation may also arise in the future in connection to the implementation of automation and robotics in the nuclear industry. Currently, there is a demand for sensors suitable for the post-accident environment, i.e., the extreme environment that occurs after nuclear accidents.

Ceramic-chromium sensors are the result of long-term research and development of metal Hall sensors at the Institute of Plasma Physics of CAS in Prague (IPP) [14]. A significant achievement of the research program is the developed steady-state magnetic field diagnostics based on the temperature and radiation resistant Hall sensors with the sensitive layer made of bismuth, which has been approved for ITER already [15]. Bismuth provides a suitable temperature operation range, high radiation resistance of the sensors as well as a relatively high output signal [15,16,17,18,19,20,21,22]. At the time of writing this article, the sensors for ITER are being calibrated, and the process of installation into the reactor will begin in 2021.

The next step in fusion research after ITER is the development of the fusion power plant DEMO. The high temperature and radiation resistant Hall sensors are proposed as a subsystem of the DEMO magnetic diagnostics performing an absolute measurement of the steady-state magnetic field [13]. The Hall sensors will contribute to the measurement of a plasma current, plasma-wall clearance, and local perturbations of the magnetic flux surfaces near the wall.

The operating temperature range of the DEMO Hall sensors will vary depending on the location of the sensors. In the case of outer-vessel sensors, it will be close to the vacuum vessel temperature, which will be approximately 200 °C [23]. In the case of inner-vessel sensors, the sensor temperature will be in the range of 300 to 520 °C [23,24]; the higher operating temperature compared to ITER exceeds the range of applicability of the bismuth sensors as bismuth melts at 271.4 °C.

Therefore, several metal sensitive layers that can operate at these temperatures have been researched [22,23]. Gold-based Hall sensors for fusion reactor application were developed at the Magnetic sensor laboratory of Lviv Polytechnic National University in Ukraine (MSL) [25,26], and antimony-based Hall sensors operating up to a temperature of 550 °C were developed at IPP [27]. Unfortunately, both gold and antimony are easily subjected to transmutation in neutron flux and are therefore not sufficiently resistant to neutron radiation [28].

Fusion power reactors will feature a strong flux of neutrons with high energy up to 14 MeV. According to the DEMO1 reference model, neutron flux of up to 1.24 × 10^18^ n/m^2^/s and neutron fluence of up to 6.08 × 10^25^ n/m^2^ after 5.2 years of operation (Phase 1 of reactor operation) are expected [28,29]. The neutron flux will cause damage to the structure of the sensitive layer material and its transmutation. Transmutation, which will change the number of free charge carriers in the material, will affect the magnitude of the Hall effect. The stability of the parameters of Hall sensors in the environment with a high neutron flux is, therefore, an important requirement associated with their operation on DEMO.

Other metals analyzed were molybdenum and chromium. It was found out that thin molybdenum nanolayers are unstable and the preparation of these sensors requires more in-depth research [23]. On the other hand, Hall sensors with a chromium nanolayer (hereafter chromium Hall sensors) have been successfully manufactured. As shown in [30], chromium also has the potential to be sufficiently resistant to neutron radiation. This paper aims to describe the first developed ceramics-chromium Hall sensors and present the results of their initial testing.

## 2. Sensor Sensitivity

The Hall sensor sensitivity *S_H_* determines the magnitude of the generated Hall voltage *V_H_*:

(1) $$V_{H}=\;S_{H}\left(T,B\right)\;I\;B,$$

where *T* is the sensor temperature, *B* is the magnetic field normal to the sensor plane, and *I* is the sensor supply current. The sensitivity is determined by the Hall coefficient *R_H_* and a sensitive layer thickness *t*:

(2) $$S_{H}\left(T,B\right)=\;\frac{R_{H}\left(T,B\right)}{t}$$

The Hall coefficient *R_H_* is a physical property of the sensitive layer material and is related to the number of free charge carriers and their mobility in the material. The Hall coefficient is generally a nonlinear function of temperature and magnetic field, but for a number of materials, some of these dependences may not occur or be negligible.

The layer thickness *t* depends on sensor design and manufacturing technology. The thin layer allows the achievement of relatively high sensitivity, but requires a suitable polished substrate and limits the supply electric current. Currently, thin sensitive layers with a thickness of nanometers to micrometers are used [23].

## 3. Ceramic-Chromium Hall Sensors

**Figure 1:**
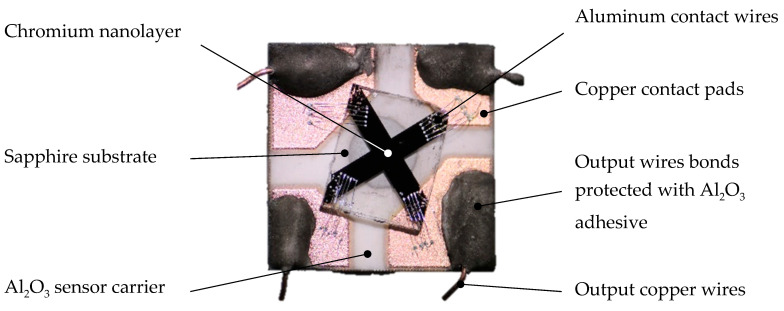
Tested chromium Hall sensor (after testing).

The first series of the chromium Hall sensors were produced using the components made of aluminum oxide Al_2_O_3_. A sensor carrier was made up of 99.6% aluminum oxide ceramics with dimensions of 10 mm × 10 mm × 0.8 mm (Figure 1).

On the sensor carrier, 50 µm thick copper contact pads were made by a thick printed copper (TPC) technology. Between the contact pads, a sapphire sensitive layer substrate with dimensions 4.5 mm × 3.5 mm × 0.5 mm was fixed with an aluminum oxide ceramic adhesive.

The nanolayer of chromium having a thickness of 50 nm had the shape of a symmetrical cross. The arms of the cross were 0.8 mm wide and 4 mm long. The nanolayer was applied to the sapphire substrate by physical vapor deposition. A Covap PVD system from Angstrom Engineering was used; the base pressure of the deposition chamber was less than 3 × 10^−5^ Pa. Chromium evaporated from a tungsten boat thermally, the deposition rate was ~0.2 nm/sec. The thickness of the layer was monitored and controlled by QCM sensors.

Aluminum contact wires with a diameter of 30 μm were ultrasonically bonded directly to the chromium layer and to the contact pads. Output copper wires were bonded to the contact pads by thermocompression and fixed with the aluminum oxide ceramic adhesive.

## 4. Methods

Two experiments and one simulation were performed to determine the basic properties of the chromium Hall sensors. The first experiment examined the temperature dependence of the sensor sensitivity. The second experiment was focused on the dependence of the sensor sensitivity on the magnetic field. The aim of the simulation was to estimate the radiation resistance of chromium based on the calculation of chromium transmutation in the neutron flux expected in the DEMO reactor.

**Figure 2:**
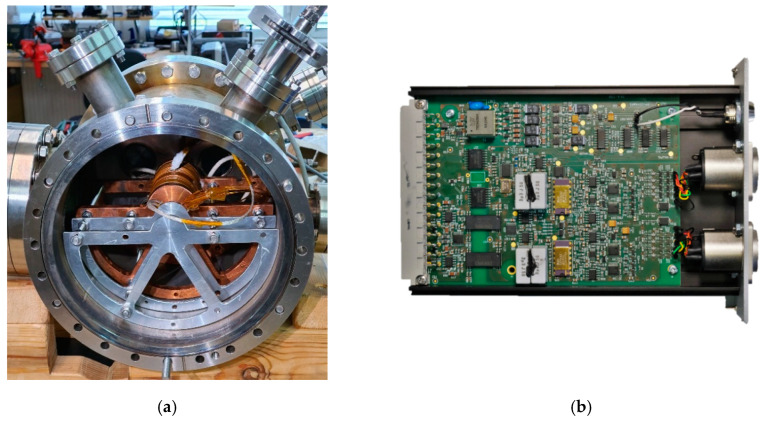
Experimental setup for high-temperature testing of the metal Hall sensors: (**a**) Vacuum oven with high-temperature solenoid with a button heater inside the solenoid; (**b**) Hall sensor controller card integrating a lock-in amplifier with a gain of 142,400.

The experiment focusing on the temperature dependence of the sensor sensitivity was performed at the Hall sensor laboratory of the Institute of Plasma Physics of CAS in Prague in a vacuum oven (Figure 2a). A stainless steel high-vacuum chamber was equipped with a ceramic resistive button heater with a heating power of up to 40 W; the heating power of 10 W was used to heat the sensor to 550 °C. The magnetic field used for testing was provided by a high-temperature solenoid located inside the chamber. The maximum operating temperature of the solenoid was 250 °C. The internal thermal shield allowed high sensor temperature to be reached without significant heating of the solenoid. The solenoid generated magnetic field pulses of 200 mT at a supply current of 100 A with an accuracy of 10 mT and precision of 0.09 mT.

The output signal of the sensor was processed by the developed Hall sensor controller (Figure 2b), which suppressed the sensor offset, planar Hall voltage, and other interfering voltages using synchronous detection and current spinning methods, and amplified the Hall voltage. The sensor was powered by 4 mA AC @ 5 kHz, the gain of the controller was 142,400. The maximum measurement error of the detected Hall voltage was 14 nV.

The tested Hall sensor was attached to the button heater and inserted into the solenoid. The heating power was maintained by feedback control. During the experiment, the temperature was raised from room temperature to 550 °C at a constant rate of 2 °C/min. The magnetic field was generated in pulses; the reason was to allow sufficient time for dissipation of the Joule heat generated within the solenoid and prevent it from overheating. Passive cooling of the solenoid was performed by a massive copper structure dissipating heat to the vacuum vessel. The pulse duration was 10 s, the idle time 2 min; during this duty cycle, the solenoid maximum temperature stabilized at 120 °C.

**Figure 3:**
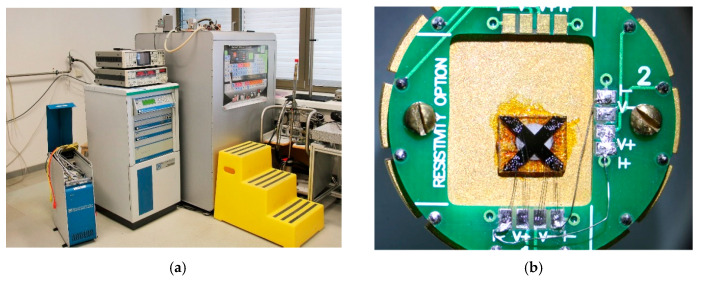
Experimental setup for high magnetic field testing of metal Hall sensors: (**a**) Super-conducting Physical Property Measurement System PPMS14; (**b**) Sensor sapphire substrate with the chromium layer installed on the PPMS puck.

The experiment focusing on the dependence of the sensor sensitivity on the magnetic field was performed in the Joint Laboratory for Magnetic Studies of the Institute of Physics of CAS and the Charles University in Prague on the superconducting Physical Property Measurement System PPMS14 from the Quantum Design Company (San Diego, CA, USA, Figure 3). The range of the operating magnetic field was from −14 T to +14 T with a maximum field error of 12 mT. The tested sensor was powered by a DC current of 4 mA. The output signal from the sensor was processed by the PPMS resistivity measurement unit with a maximum measurement error of 20 nV. The maximum test temperature in PPMS was 126.8 °C (400 K).

The sensor substrate with the sensitive layer without the ceramic sensor carrier was installed on a PPMS puck (Figure 3), which was then inserted into a superconducting magnet. The measurement was performed according to the program script by scanning sensitive layer resistivity in the range of the magnetic field from −14 T to +14 T in steps of 0.5 T at a sensor temperature of 20 °C and 120 °C. The wire connection enabled the measurement of transverse and longitudinal resistivity.

**Figure 4:**
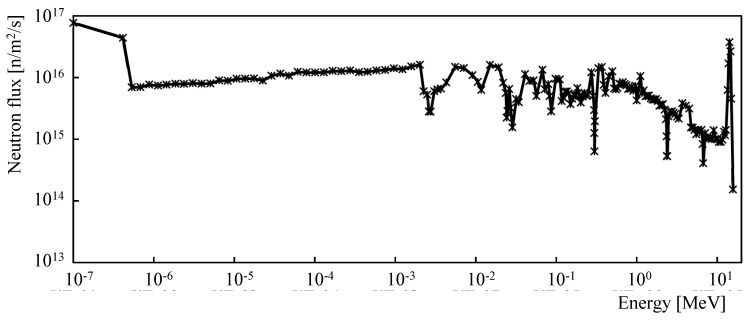
Neutron energy spectrum (175 energy groups) at the in-vessel divertor location of DEMO1 reactor used for the simulation [28,31].

The simulation of the neutron-induced transmutation of chromium was performed following the earlier analysis reported in [28]. A neutron spectrum divided into 175 energy groups [31] determined by the MCNP6 code was used in the simulation. For this analysis, the neutron spectrum at the in-vessel divertor location was conservatively selected due to the strongest neutron flux (Figure 4). The simulation of transmutation was performed using the FISPACT-II code [32]; the detailed description of the code, parameter settings, and calculation methods are given in [28]. The composition of the chromium sensitive layer was evaluated every 24 h of the simulated time until the DEMO Phase 1 operation time was reached. The same calculation methods and settings used as in the previous analysis make it possible to compare the extent of chromium transmutation with the extent of transmutation of already analyzed materials.

## 5. Results

**Figure 5:**
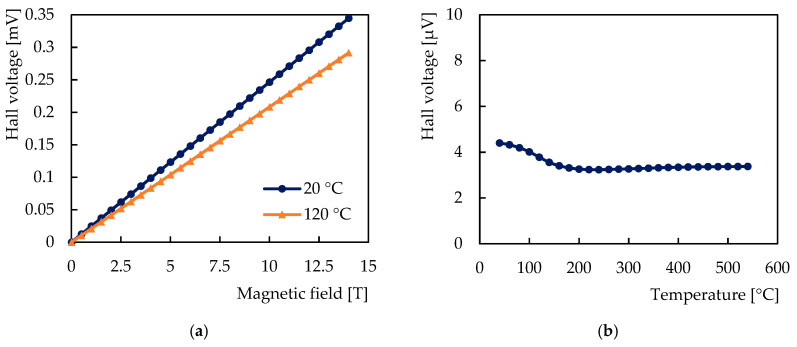
Hall voltage of the tested sensor with chromium sensitive nanolayer: (**a**) Dependence on the magnetic field at 20 °C and at 120 °C; (**b**) Dependence on the temperature in the magnetic field of 200 mT.

**Figure 6:**
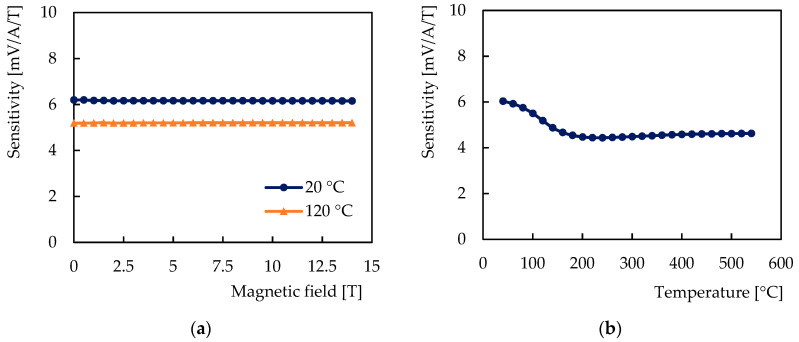
Sensitivity of the tested sensor with chromium sensitive nanolayer: (**a**) Dependence on the magnetic field at 20 °C and at 120 °C; (**b**) Dependence on the temperature in the magnetic field of 200 mT.

**Figure 7:**
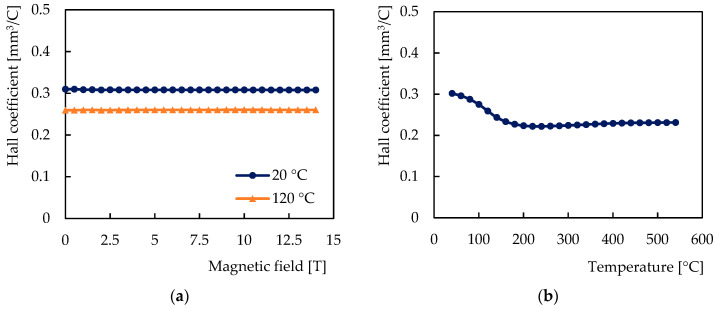
Hall coefficient of chromium of the sensitive nanolayer: (**a**) Dependence on the magnetic field at 20 °C and at 120 °C; (**b**) Dependence on the temperature in the magnetic field of 200 mT.

The observed dependences of the Hall voltage on the magnetic field and the temperature are shown in Figure 5. Figure 6 shows the dependences of the sensor sensitivity on the magnetic field and the temperature, and Figure 7 shows the behavior of the Hall coefficient of the sensitive layer chromium. Figures show the dependences on the magnetic field for two sensor temperatures: 20 °C and 120 °C, and the dependences on the temperature from 20 °C to 550 °C in the magnetic field of 200 mT.

The dependence of the Hall voltage on the magnetic field was found to be linear, the sensitivity and the Hall coefficient were found to be independent of the magnetic field over the entire range of the test field. The relative average deviation of the values of the sensitivity and the Hall coefficient was determined to be 0.081%, which is below the relative measurement accuracy of 0.086% of the full range corresponding to the above accuracy of the PPMS experimental setup of 12 mT.

The observed temperature behavior can be divided into three temperature ranges according to the temperature coefficient: from room temperature to 240 °C, from 240 °C to 400 °C, and from 400 °C to 550 °C.

In the temperature range from 20 °C to 240 °C, the sensitivity decreased by 28.4% from 6.20 mV/A/T to 4.44 mV/A/T. Its temperature coefficient was negative in the range from −0.005 to −0.299%/°C. In the temperature range from 240 °C to 400 °C, the sensitivity increased by 3.3% from 4.44 mV/A/T to 4.58 mV/A/T, and the temperature coefficient was less than 0.027%/°C. In the temperature range from 400 °C to 550 °C, the sensitivity increased by 1% from 4.58 mV/A/T to 4.63 mV/A/T, and the temperature coefficient was less than 0.014%/°C.

The Hall coefficient features the same curve as in the case of the sensitivity, and its magnitude was found to be 3.1 × 10^−10^ m^3^/C at 20 °C and 2.3 × 10^−10^ m^3^/C at 500 °C.

**Figure 8:**
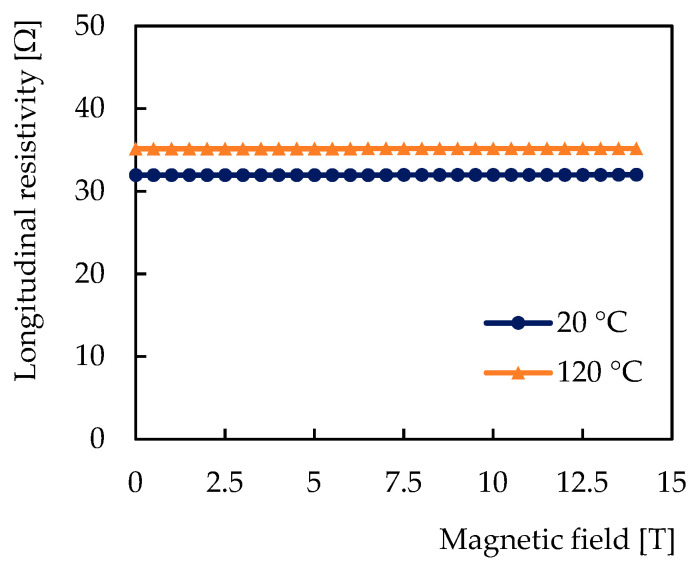
Dependence of the longitudinal resistivity of the sensitive layer on the magnetic field at 20 °C and at 120 °C.

In the experiment, we also focused on the dependence of the longitudinal resistance on the magnetic field; however, no measurable magnetoresistance appeared up to 14 T as shown in Figure 8.

**Figure 9:**
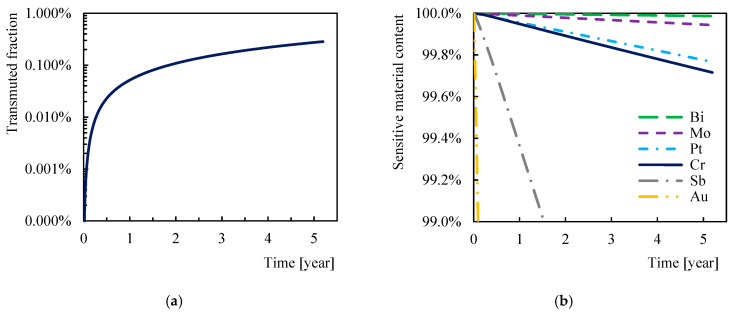
Transmutation of chromium under the neutron flux of the DEMO1 fusion power reactor during the operation Phase 1 (5.2 years): (**a**) Transmuted fraction of the chromium sensitive layer; (**b**) Comparison of the rate of transmutation of some metals and semi-metals.

The simulation of the chromium neutron-induced transmutation presented in Figure 9a shows the expected gradual increase in the number of transmuted atoms, which reduces the chromium content in the sensitive layer. Chromium has been found resistant to neutron transmutation: after performing DEMO Phase 1 featuring 5.2 operational years; the transmutation fraction of chromium reached less than 0.27% at the most exposed sensor location with a neutron fluence of 6.08 × 10^25^ n/m^2^. Chromium transmuted almost entirely to vanadium, other elements such as manganese or titanium were formed in negligible amounts (<0.005%).

As shown in Figure 9b, the chromium transmutation rate was found to be similar to platinum, bismuth, and molybdenum, while antimony and gold transmute much faster [28].

## 6. Discussion

The Hall coefficient *R_H_* of chromium of the tested sensitive nanolayer was found to be positive and weakly temperature-dependent in the range shown in Figure 7 and its value never drops below 2.2 × 10^−10^ m^3^/C. In the pursuit of theoretical understanding of such finding, the first fact to notice is that chromium orders antiferromagnetically below 35 °C (character of magnetic order is complicated [33] and not important for the subsequent discussion). Short-range correlations between magnetic moments persist even above the ordering temperature, so the decrease of *R_H_* up to approximately 200 °C can likely be attributed to the gradual disappearance of magnetic order.

To model *R_H_* in the paramagnetic (high-temperature) phase, we took two steps. First, using a linearized augmented-plane wave ab initio method [34] and semiclassical formalism [35], we calculated the conductivity tensor of a chromium monocrystal (body-centered cubic structure with a lattice constant 0.2885 nm was assumed). These calculations suffer from a large numerical error originating probably from the second derivatives of band dispersion *E(k)* appearing in Equation (10) of Ref. [35]. However, the values of *R_H_* obtained in this way by density functional theory (DFT) approach the mentioned level 2 × 10^−10^ m^3^/C and can thus be regarded as consistent with experimental data obtained on polycrystalline samples (see Figure 7). Second, we made an analytical estimate:

(3) $$R_{H}\approx \frac{1}{ne}\;\left(1-\frac{1}{8}x^{4}\right),x=\frac{k_{B}T}{2E_{F}}\;$$

for ideal electron gas of density *n* related to the Fermi level as $E_{F}\propto n^{2/3}$. This result can be obtained by inserting free-electron dispersion $E\left(k\right)=\hbar^{2}k^{2}/2m$ into Equation (15) of Ref. [35], and an analytical evaluating the integral after expansion in powers of *x* (the Sommerfeld expansion) was carried out. Equation (3) is valid *o(x*^4^*)*. Assuming the typical Fermi level of a metal to be of the order of 1 eV which corresponds to the band width of transition metals, elevated temperatures in our work correspond to *x* in the range between 0.01 and 0.1. Equation (3) then implies negligible corrections to *R_H_*, and it is, therefore, understandable that the temperature dependence of *R_H_* shown in Figure 7 is very weak once the magnetic order is completely destroyed. We note that upon deriving Equation (3), the assumption of constant relaxation time was taken; hence, it is not surprising that certain other metals may exhibit an appreciable temperature dependence of the Hall coefficient.

In terms of the magnitude of the Hall coefficient of chromium, the value of 3.1 × 10^−10^ m^3^/C found at room temperature is close to values reported earlier, e.g., [9] (3.8 × 10^−10^ m^3^/C as given in Table 1). The difference may be due to the specific (polycrystalline) structure of the sensitive layer of the tested sensor.

**Figure 10:**
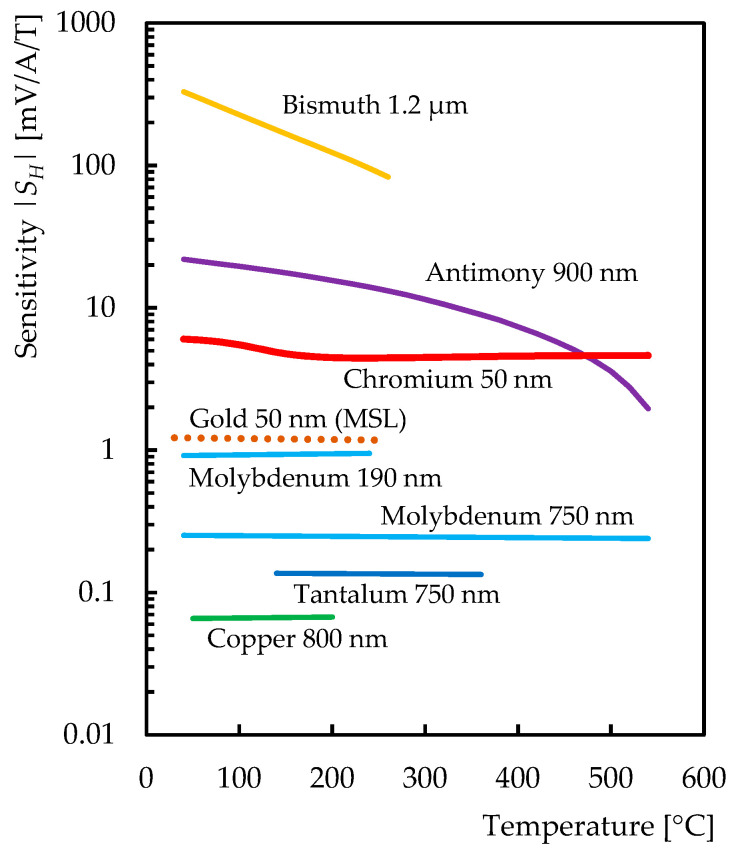
Sensitivity of the semimetal and metal Hall sensors tested so far [23].

A comparison with the semimetal (Bi, Sb) and metal (Au, Mo, Ta, Cu) Hall sensors tested so far [23] is presented in Figure 10. In agreement with Equation (3), all metal Hall sensors feature only a weak temperature-dependence of the sensitivity (which is proportional to the Hall coefficient). The chromium Hall sensor achieved the highest sensitivity of all the sensors tested at temperatures above 480 °C.

In the operating temperature range of 300 to 520 °C of the Hall sensors intended for the DEMO fusion reactor, the sensor sensitivity changed from 4.49 mV/A/T to 4.62 mV/A/T with the temperature coefficient in the range of 0.004–0.027%/°C. Assuming stabilization of the sensor operating temperature in the range of 10 °C [23], temperature changes will cause a magnetic field measurement error less than 0.14% at temperatures from 300 °C to 420 °C, and 0.02% at temperatures from 420 °C to 520 °C.

Simulation of neutron-induced transmutation showed high chromium resistance, but it is difficult to predict how the gradual transmutation of up to 0.27% chromium to vanadium will change the Hall effect. Vanadium has a lower Hall coefficient than chromium (7.6 × 10^−11^ m^3^/C at room temperature [9]); unfortunately, the measurement of the Hall coefficient of the corresponding alloys at relevant temperatures is not available. Furthermore, the radiation environment leaves a bigger impact than just transmutation alone. Therefore, it is necessary to test the radiation resistance of the sensors in a real environment setting with strong radiation. Irradiation tests of the sensors with a gradual increase in neutron fluence will be performed in the LVR-15 nuclear reactor at the Research Center Rez in the Czech Republic.

Metal Hall sensors will also be a part of the diagnostics of the new COMPASS-U tokamak, which is under construction at IPP. The COMPASS-U tokamak will operate at a high magnetic field of 5 T and a first wall temperature of up to 500 °C [36].

## 7. Conclusions

The new ceramic-chromium Hall sensors with the 50 nm thick chromium sensitive layer were successfully tested for temperatures up to 550 °C and in the magnetic field up to 14 T. The sensitivity of the sensor was observed weakly dependent on the temperature at temperatures above 240 °C with the average temperature coefficient of 0.014%/°C and independent of the magnetic field. The transmutation analysis indicated a high resistance of the chromium-sensitive layer to neutron radiation; after 5.2 operational years, the transmuted fraction of the chromium sensitive layer was less than 0.27% at the most exposed sensor location behind the DEMO reactor divertor cassette wherein a neutron fluence reached 6.08 × 10^25^ n/m^2^.

In summary, the ceramic-chromium Hall sensors have the potential to be suitable magnetic sensors for environments with high temperatures and neutron radiation and candidates for the steady-state magnetic field diagnostics of the DEMO fusion reactors.

## Figures and Tables

**Table 1:** Hall coefficients at room temperature.

Sensitive Material		Hall Coefficient [mm^3^/C]
InSb	[3]	350,000.00
Bi	[7]	−540.00
Sb	[8]	−21.00
Cr	[9]	0.38
Mo	[9]	0.13
Au	[9]	−0.07
Cu	[9]	−0.05
